# A comparative analysis of near-infrared image colorization methods for low-power NVIDIA Jetson embedded systems

**DOI:** 10.3389/fnbot.2023.1143032

**Published:** 2023-04-24

**Authors:** Shengdong Shi, Qian Jiang, Xin Jin, Weiqiang Wang, Kaihua Liu, Haiyang Chen, Peng Liu, Wei Zhou, Shaowen Yao

**Affiliations:** ^1^Engineering Research Center of Cyberspace, Yunnan University, Kunming, Yunnan, China; ^2^School of Software, Yunnan University, Kunming, China; ^3^Guangxi Power Grid Co., Ltd., Nanning, China

**Keywords:** near-infrared image, image colorization, Jetson, performance evaluation, embedded systems

## Abstract

The near-infrared (NIR) image obtained by an NIR camera is a grayscale image that is inconsistent with the human visual spectrum. It can be difficult to perceive the details of a scene from an NIR scene; thus, a method is required to convert them to visible images, providing color and texture information. In addition, a camera produces so much video data that it increases the pressure on the cloud server. Image processing can be done on an edge device, but the computing resources of edge devices are limited, and their power consumption constraints need to be considered. Graphics Processing Unit (GPU)-based NVIDIA Jetson embedded systems offer a considerable advantage over Central Processing Unit (CPU)-based embedded devices in inference speed. For this study, we designed an evaluation system that uses image quality, resource occupancy, and energy consumption metrics to verify the performance of different NIR image colorization methods on low-power NVIDIA Jetson embedded systems for practical applications. The performance of 11 image colorization methods on NIR image datasets was tested on three different configurations of NVIDIA Jetson boards. The experimental results indicate that the Pix2Pix method performs best, with a rate of 27 frames per second on the Jetson Xavier NX. This performance is sufficient to meet the requirements of real-time NIR image colorization.

## 1. Introduction

In surveillance and vehicle driving scenes (Ni et al., 2022), color image sensors are preferred because their images are close to human visual perception. However, visible images have obvious limitations related to lighting conditions (Yu et al., 2022) and the color of an object’s surface (Liao et al., 2022). However, NIR sensors are usually used in night vision and low-illumination scenes because they provide more useful information than visual sensors (Jin et al., 2017). An NIR image is a shaded gray image, which is not in line with human visual habits; so, it is preferable to colorize it, enhancing its color and texture information. Colorized images can improve an observer’s ability to assess a scene and increase the efficiency of target detection. The problem of image colorization lies in generating a plausible visible image from only an NIR image (Sun et al., 2019). Thus, NIR image colorization aims to generate a reasonable visible image from an NIR image while preserving the texture in the NIR domain so that the coloring of the converted visible image looks natural.

In the common gray image colorization domain, chromaticity is the only feature that needs to be calculated because the input gray image provides brightness levels. However, the colorized results of NIR images are usually fuzzy and lack high-frequency scene details. Therefore, it is necessary to test the common gray image colorization methods to determine whether they are suitable for NIR image colorization on embedded systems (Liang et al., 2021).

Deep learning models usually require many computing resources (Qin et al., 2020; Fortino et al., 2021), which are deployed on the cloud server. The large amount of data collected by video surveillance equipment needs to be processed by the cloud server (Ma et al., 2018) so that the deep learning model of image processing is affected by network delay (Zhang et al., 2020) or shutdown. Since a deep learning model can be deployed on edge devices that process data in real-time, there is no need to connect the cloud computing platform to process the data from an edge of the network (Han et al., 2020). This would reduce latency and bandwidth costs, improving availability and protecting data privacy and security (Shi et al., 2016). For example, many researchers deploy target detection (Zhao et al., 2019) and visual tracking (Cao et al., 2022) to the edge device for testing and striving for real-time processing.

There has been considerable research that evaluated the effectiveness of various image processing methods (Jin et al., 2017; Liu et al., 2020; Huang et al., 2022). However, most colorization techniques have not been tested for edge devices, and there is no widely recognized system for evaluating these methods on edge devices. However, image colorization has many potential applications on edge devices (Liu et al., 2022). Our study designed an evaluation system to examine the performance of current methods on edge devices. Eleven image colorization methods were tested for NIR image datasets on the Jetson AGX Xavier, Jetson Xavier NX, and Jetson Nano devices. Seven indexes were selected in analyzing the experimental results, and the results using each index were tabulated for evaluating the performance of each method on an edge device.

The contributions of this work are as follows:

We analyzed current image colorization methods to provide guidance in their practical application.

We deployed and tested image colorization methods on three different edge devices and analyzed their resource utilization and energy consumption.

This work inferred general rules and determined key points requiring attention in evaluating the performance of test methods. These were based on the performance of current image colorization methods on edge devices, focusing on resource occupancy, energy consumption, and image quality metrics.

Section 1 summarizes the status of current research on NIR image colorization and the deployment of models on edge devices. Section 2 introduces the structure and operation of the proposed evaluation system and explains why the tested models were chosen. The edge devices used and the evaluation metrics are also described in detail. Experiments on three edge devices and the RGB-NIR scene dataset (Brown and Süsstrunk, 2011) are described in Section 3. Section 4 presents the conclusions of our work and possible directions of future development in this research.

## 2. Materials and methods

**FIGURE 1:**
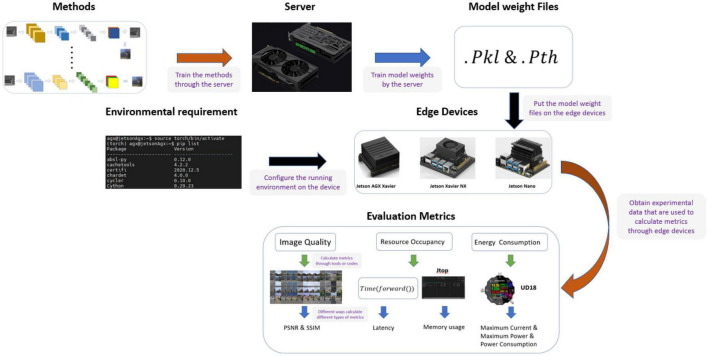
Architecture of the evaluation system.

In this work, we designed a system for evaluating the performance of an image colorization method on edge devices, as shown in Figure 1. We selected 11 classical image colorization methods based on their network structures, and we briefly introduce these models’ structures here. We trained these models using the RGB-NIR scene dataset (Brown and Süsstrunk, 2011) on a server equipped with an RTX3060 GPU to obtain the corresponding model weight files. Then, according to the development of current embedded devices, Nvidia Jetson series edge devices were selected. The Jetson AGX Xavier, Jetson Xavier NX, and Jetson Nano offer high, middle, and low performance levels, respectively. When configuring the software environment of the edge device, we chose the system with the same version number from NVIDIA, which ensures that the software environment for the three edge devices is as similar as possible. According to the environmental requirements of different models, we configured the running environment for each device and compiled the ARM Python package suitable for the particular device. Then, we uploaded the model weight files from the server to each edge device. To better compare the various methods’ performance on edge devices, we selected seven evaluation metrics for the experiment. Finally, we analyzed the experimental data and summarized the results of the experiments presented in this paper.

### 2.1. Image colorization methods

In recent years, methods based on convolutional neural networks (CNNs) have been used extensively in computer vision. ResNet (He et al., 2016) and deep convolution generated adversarial networks (DCGANs) (Radford et al., 2015) are two types of neural networks that have become popular recently. Finding meaningful information in the image is an essential problem in machine vision and image processing research. Attention mechanisms have also attracted the interest of researchers in image processing (Zhu et al., 2023). Many image colorization methods have been proposed based on these structures (Huang et al., 2022).

#### 2.1.1. Convolutional neural network

**FIGURE 2:**
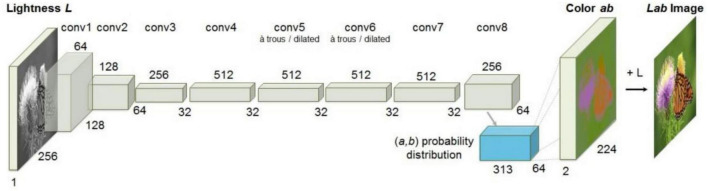
Architecture of CICZ (Zhang et al., 2016).

CICZ (Zhang et al., 2016) is an automatic image colorization method that transforms the colorizing problem into a classification problem by quantifying the color space and combining the method of category balancing, as shown in Figure 2. The encoder-decoder structure is adopted. The L channel of the grayscale image is input to predict the a and b channels of the image, and then, the colorized result is obtained.

**FIGURE 3:**
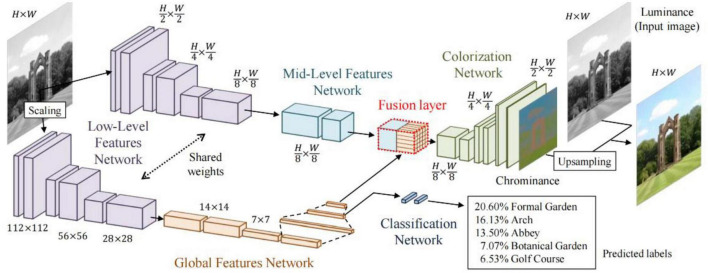
Architecture of ELGL (Iizuka et al., 2016).

ELGL (Iizuka et al., 2016) is a fully automatic image colorization method that combines global information and local features, as shown in Figure 3. The method first extracts shared low-level features from the image and then uses these features to obtain global image features and middle-level image features. Next, the shallow and global features are fused through the fusion layer, which inputs the result to the colorization network and outputs the final chrominance information.

#### 2.1.2. Wasserstein generated adversarial network

**FIGURE 4:**
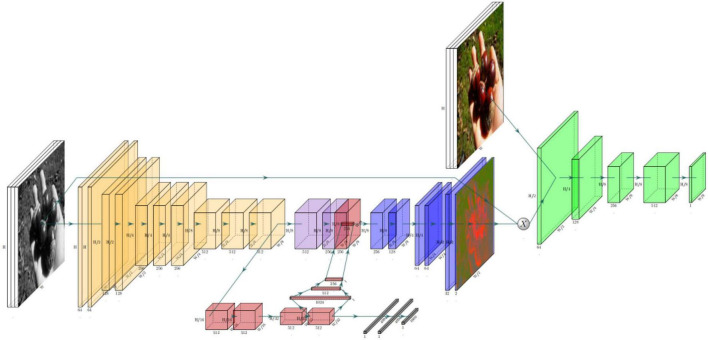
Architecture of ChromaGAN (Vitoria et al., 2020).

ChromaGAN (Vitoria et al., 2020) is an adversarial learning colorization method that infers the chromaticity of a given grayscale image according to semantic clues. In the adversarial network-based method, a three-term loss function combining color, perceptual information, and semantic category distribution was proposed. A self-supervised strategy is used to train the model. The discriminator is based on Markovian architecture [PatchGAN (Isola et al., 2017)]. Figure 4 shows the method’s block diagram.

**FIGURE 5:**
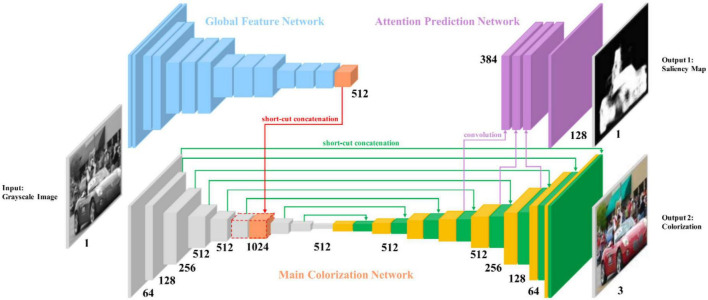
Architecture of SCGAN (Zhao et al., 2020)’s generator.

**FIGURE 6:**
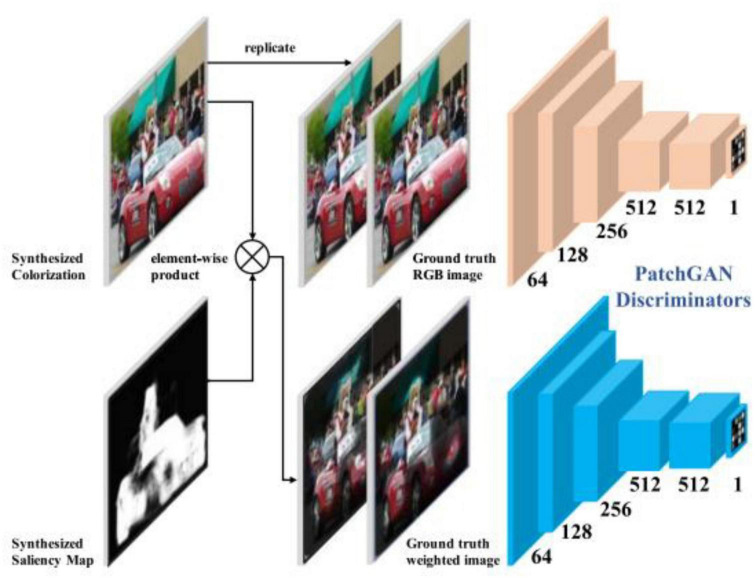
Architecture of SCGAN (Zhao et al., 2020)’s discriminator.

SCGAN (Zhao et al., 2020) is an automatic saliency map-guided colorization method with a generative adversarial network. It combines predictive colorizing and saliency maps to minimize semantic confusion and color bleeding in the colorized image, as shown in Figure 5. The global features of the pre-trained VGG-16-Gray network were embedded in the color encoder. Branches of the color decoder are used to predict saliency maps as proxy targets. Then, the method uses two hierarchical discriminators to distinguish between the generated colorized result and saliency maps, as shown in Figure 6.

#### 2.1.3. Conditional generated adversarial network

**FIGURE 7:**
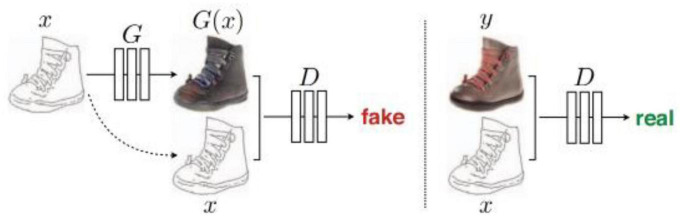
Architecture of Pix2Pix (Isola et al., 2017).

Pix2Pix (Isola et al., 2017) is based on the idea of a conditional generated adversarial network (CGAN). Generator G uses the U-Net structure. The input contour map *x* is encoded and decoded into a real image. The discriminator D uses the condition discriminator PatchGAN proposed by the author himself. The function of discriminator D is to judge the generated image as false and the real image as true under the condition of the contour map *x*. Figure 7 shows the structure of Pix2Pix.

**FIGURE 8:**
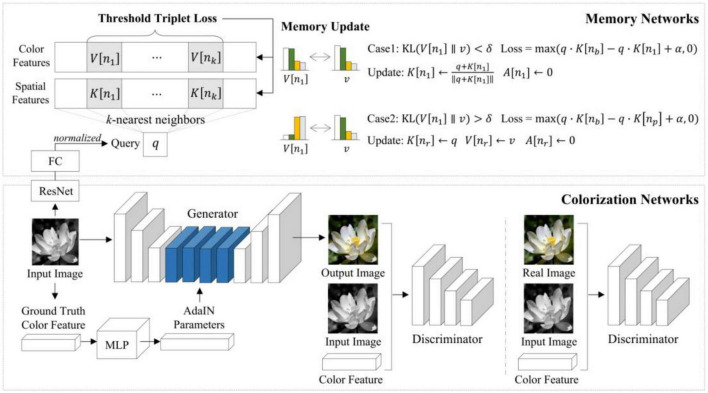
Architecture of MemoPainter (Yoo et al., 2019).

MemoPainter (Yoo et al., 2019) is a novel storage memory-enhanced colorizing model that obtains the given color information in the training set with the memory network by querying to guide colorizing. This model can generate high-quality colorized images from limited data and proposes a novel threshold triplet loss, which can complete unsupervised training of storage networks under classless labels. MemoPainter’s architecture is shown in Figure 8.

**FIGURE 9:**
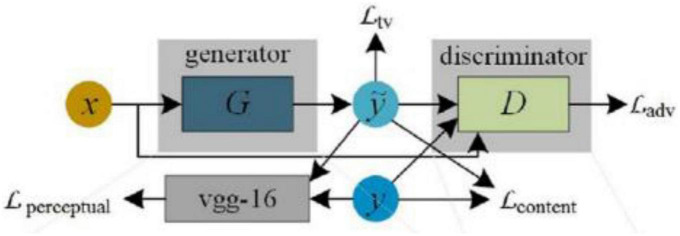
Architecture of TIC-CGAN (Kuang et al., 2020).

TIC-CGAN (Kuang et al., 2020) uses a detail-preserving coarse-to-fine generator to learn transformation mapping, as shown in Figure 9. The method proposes a composite loss function that integrates content, adversarial, perceptual, and total variation loss. Content loss is used to restore global image information, and the other three losses synthesize local realistic textures.

#### 2.1.4. Cycle-consistent adversarial network

**FIGURE 10:**
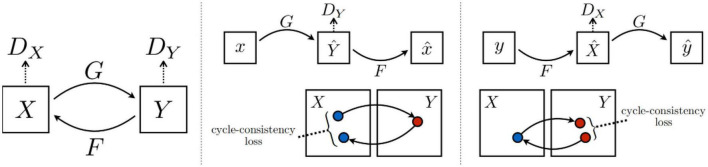
Architecture of CycleGAN (Zhu et al., 2017).

CycleGAN (Zhu et al., 2017) is an unsupervised GAN. Its main idea is to train two pairs of generator-discriminator models (two mapping functions G: X—> Y and F: Y—> X) to convert images from one domain to another. In this process, two cycle-consistency losses are introduced to ensure that the generator does not convert an image from one domain to another that is entirely unrelated to the original image. The architecture of CycleGAN is shown in Figure 10.

**FIGURE 11:**
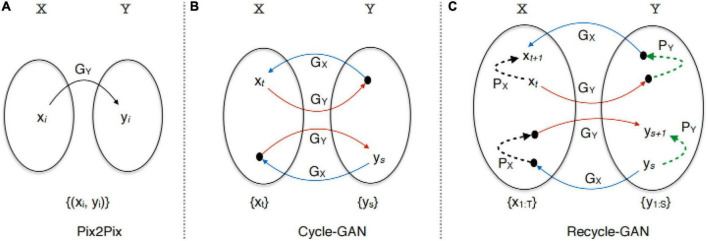
Architecture of RecycleGAN (Bansal et al., 2018).

RecycleGAN (Bansal et al., 2018) is an unsupervised data-driven method for video redirection that combines spatial and temporal information and adversarial loss for content translation and style retention for video redirection. The method proves that under different conditions, the use of time information provides more constraints for optimizing the transformation from one domain to another, which helps to obtain better local minima. The combination of temporal and spatial constraints helps to learn the style characteristics of a given domain. The difference in design between this method and CycleGAN (Zhu et al., 2017) and Pix2Pix (Isola et al., 2017) is shown in Figure 11.

**FIGURE 12:**
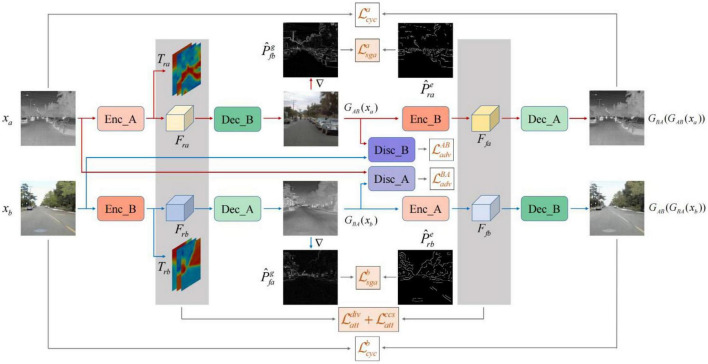
Architecture of PearlGAN (Luo et al., 2022).

PearlGAN (Luo et al., 2022) is a GAN based on top-down attention and gradient alignment. First, a top-down guided attention module and an elaborate attentional loss reduce semantic coding ambiguity during translation. Then, the model introduces a structured gradient alignment loss to encourage edge consistency between transmissions. The internal structure of PearlGAN is shown in Figure 12.

**FIGURE 13:**
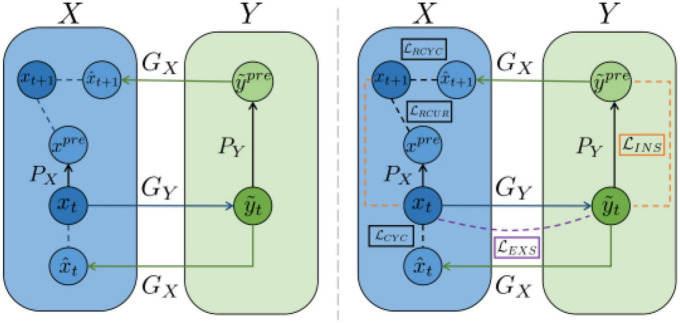
Architecture of I2V-GAN (Li et al., 2021).

I2V-GAN (Li et al., 2021) is an infrared-to-visible video conversion method that generates fine-grained and spatiotemporally consistent visible video from a given unpaired infrared video, as shown in Figure 13. The model utilizes adversarial constraints to generate a synthetic frame similar to the real frame and then introduces the circular consistency of perceptual loss for effective content transformation and style preservation. Finally, it utilizes the similarity constraints between and within domains to enhance the content and motion consistency of space and time-space at the fine-grained level.

### 2.2. Edge devices

While the Raspberry Pi offers low power consumption and energy-saving performance, NVIDIA Jetson platforms have a higher GPU speed, leading to better deep learning inference performance. The security and reliability of the NVIDIA Jetson series make it possible to deploy deep learning models in harsh environments; hence, the Jetson series of edge devices have been used in many industrial fields. The Jetson platform is compatible with the Jet Pack software development kit, which includes libraries for deep learning, such as computer vision and accelerated computing. By using the same version of the NVIDIA official system, we can maintain consistency in the experimental environment to a certain degree. Thus, we test the performance of different models on Jetson AGX Xavier, Jetson Xavier NX, and Jetson Nano devices that belong to three edge devices of high, middle, and low-performance levels. It is appropriate to compare the performance of different models under the constraints of different hardware conditions.

#### 2.2.1. Jetson AGX Xavier

Jetson AGX Xavier is a 30 W GPU workstation from NVIDIA that was launched in December 2018. Its CPU is eight-core ARM NVIDIA Carmel, the GPU is NVIDIA Volta architecture with 512 NVIDIA CUDA cores, and the memory is 32 GB LRDDR4x. Jetson AGX Xavier provides good memory bandwidth and computing performance. It has a computing speed of up to 32 TOPS (30 W) in deep learning and computer vision tasks. For image processing tasks, real-time effects can be achieved on some models (Mazzia et al., 2020; Jeon et al., 2021).

#### 2.2.2. Jetson Xavier NX

Jetson Xavier NX is a mid-end product launched by NVIDIA in November 2019. Its CPU is 6-core ARM NVIDIA Carmel, the GPU is NVIDIA Volta architecture with 384 NVIDIA CUDA cores, and the memory is eight GB LRDDR4x. Due to the Volta architecture, it has a server-level performance of up to 21 TOPS (15 W) or 14 TOPS (10 W). For image processing tasks, Jetson Xavier NX already offers the performance requirements of most models (Jeon et al., 2021).

#### 2.2.3. Jetson Nano

Jetson Nano is an entry-level product launched by NVIDIA in March 2019. Its CPU is four-core ARM A57, the GPU is NVIDIA Maxwell architecture with 128 NVIDIA CUDA cores, and the memory is four GB LRDDR4, which supports switching between 5 W and 10 W modes. The Jetson Nano has the lowest performance in the series at only 0.5 TFLOPS, but it also has the lowest price and power consumption, making it more suitable for use in less-demanding edge scenes. The Jetson Nano is unsuitable for infrared image colorization, mainly playing a comparative role in the experiment (Mazzia et al., 2020).

### 2.3. Evaluation metrics

In research on deploying deep learning methods in edge devices, the allocation of computing resources is a crucial concern. The choice of resources varies depending on the specific scenario. Computing resources such as CPU, GPU, and memory are considered for computing-sensitive tasks (Toczé and Nadjm-Tehrani, 2018). Storage and communication resources such as IO, hard disk, spectrum, and bandwidth are considered for data-sensitive tasks (Toczé and Nadjm-Tehrani, 2018).

The evaluation metrics selected in this work include Peak Signal to Noise Ratio (PSNR), Structural Similarity (SSIM), latency, memory usage, maximum current, maximum power, and power consumption. We have considered CPU occupancy, but in the actual test process, the occupancy rate is difficult to evaluate as a metric because of its multi-core architecture.

#### 2.3.1. Image quality

Peak Signal to Noise Ratio is generally used between the maximum signal and background noise. Usually, after image processing, the processed image *x_1_* will be different from the original image *x_2_*. To measure the quality of the processed image, we usually refer to the PSNR value to measure whether a processing program is satisfactory. PSNR’s formula is shown in Equation 1. $M\,A\,X_{x_{1}}^{2}$ represents the maximum pixel value of the processed image *x*_1_. The size of the processed image *x_1_* and original image *x_2_* is m*n. PSNR can be calculated as follows:


(1) $$P\,S\,N\,R=10^{*}\,\log_{10}\left(\frac{M\,A\,X_{x_{1}}^{2}}{{\frac{1}{m^{*}\,n}}^{*}\,\sum_{i=1}^{m}\sum_{j=1}^{n}{\left[x_{2}\,\left(i,j\right)-x_{1}\,\left(i,j\right)\right]}^{2}}\right).$$

Structural Similarity is a metric that considers luminance, contrast, and structure. The SSIM value of two images is calculated using the original image *x_2_* and the processed image *x_1_*. SSIM can measure the degree of distortion and the similarity between the two images. SSIM ranges from –1 to 1. When two images are the same, the SSIM value is 1. SSIM’s formula is shown in Equation 2. *l*(*x*_2_,*x*_1_) represents the luminance contrast function. *c*(*x*_2_,*x*_1_) represents the contrast function. *s*(*x*_2_,*x*_1_) represents the structural contrast function. μ_*x*_2__ and μ_*x*_1__ represent the averages of *x_2_* and *x_1_*, respectively. σ_*x*_2__ and σ_*x*_1__ represent the variances of *x_2_* and *x_1_*, respectively. σ_*x*_2_*x*_1__ represents the covariances of *x_2_* and *x_1_*. θ_1_, θ_2_, and θ_3_ are designed with three constants to avoid zero denominators. SSIM is given by


(2) $$S\,S\,I\,M\,\left(x_{2},x_{1}\right)={\left[l\,\left(x_{2},x_{1}\right)\right]}^{\alpha }\,{\left[c\,\left(x_{2},x_{1}\right)\right]}^{\beta }\,{\left[s\,\left(x_{2},x_{1}\right)\right]}^{\gamma },w\,h\,e\,r\,e$$

(3) $$l\,\left(x_{2},x_{1}\right)=\frac{2\,\mu_{x_{2}}\,\mu_{x_{1}}+\theta_{1}}{\mu_{x_{2}}^{2}+\mu_{x_{1}}^{2}+\theta_{1}},$$

(4) $$c\,\left(x_{2},x_{1}\right)=\frac{2\,\sigma_{x_{2}}\,\sigma_{x_{1}}+\theta_{2}}{\sigma_{x_{2}}^{2}+\sigma_{x_{1}}^{2}+\theta_{2}},a\,n\,d$$

(5) $$s\,\left(x_{2},x_{1}\right)=\frac{\sigma_{x_{2}\,x_{1}}+\theta_{3}}{\sigma_{x_{2}}\,\sigma_{x_{1}}+\theta_{3}}.$$

#### 2.3.2. Resource occupancy

By comparing the latency of different models, a suitable model is selected to deploy in different industrial application scenarios (Cao et al., 2022). Meanwhile, the memory of edge devices is a scarce resource because multiple models with different purposes may need to be deployed. By clarifying the memory usage of different models, we can select a suitable model without affecting the deployment of other models.

Latency refers to the average time consumed per image when the model colorizes the image continuously. Because the time consumed is the same as different models have the same operation when reading and saving images, we only calculate the time consumed in generating the colorized image [*forward*()]. We tested 20 NIR images 100 times to calculate the accurate latency and then averaged them. The formula to calculate latency is shown in Equation 6. *a* represents the number of different images used to calculate the latency. *b* represents the number of times the same image runs *forward*(). *Time*() represents the time calculation function. Frames Per Second (FPS) is also used in this article to represent inference speed, as shown in Equation 7. The formulas are as follows:


(6) $$Latency=\frac{1}{a}\overset{a}{\underset{i=1}{\sum }}\;\frac{Time(forward()*b)}{b}\text{and}$$

(7) $$F\,P\,S=\frac{1}{L\,a\,t\,e\,n\,c\,y}.$$

Memory usage refers to the occupied memory monitored by the system process Jtop during the model test. We use RAM to denote the occupied memory in the experiment, including the video memory of the Jetson device, which is also calculated as a part of memory. In contrast, the video memory of the server with RTX 3,060 is calculated separately; so, when comparing the results, the sum of the memory usage and the video memory usage of the server is calculated. To test the accurate memory usage of the colorizing image, we continue to colorize the image for 180 s. The test results are the increase in memory usage from reading an NIR image to outputting a colorized image.

#### 2.3.3. Energy consumption

In laboratory studies, we usually do not consider the energy consumed by the model operation. In the actual application scenario, users take the energy consumption problem seriously. Therefore, recording the model’s energy consumption when deployed on edge devices makes sense.

The maximum current (Imax) refers to the maximum current recorded. The maximum power (Pmax) is the product of the maximum current and voltage. Power consumption (PC) refers to the total power consumption of the model running on the edge device for a certain time. The UD18 detector measures these three metrics during the model test. To test the accurate data, we need only to test the function of colorizing images and can continue to colorize the image for 180 s. It measures the process of end-to-end inference, from reading an NIR image to outputting a colorized image.

## 3. Results and analysis

We trained the different methods using a computer with an AMD Ryzen7 5800H 3.2 GHz CPU and one NVIDIA Geforce RTX 3060 GPU. We compared the following methods [CICZ (Zhang et al., 2016), ELGL (Iizuka et al., 2016), ChromaGAN (Vitoria et al., 2020), SCGAN (Zhao et al., 2020), Pix2Pix (Isola et al., 2017), MemoPainter (Yoo et al., 2019), TIC-CGAN (Kuang et al., 2020), CycleGAN (Zhu et al., 2017), RecycleGAN (Bansal et al., 2018), PearlGAN (Luo et al., 2022), and I2V-GAN (Li et al., 2021)] on three different edge devices based on the selected metrics.

### 3.1. Experimental dataset

We used the RGB-NIR scene dataset (Brown and Süsstrunk, 2011), which contains 477 image pairs with a resolution of 1,024 × 680 captured from nine scene categories. Image scene categories were villages, fields, forests, indoors, mountains, ancient buildings, streets, cities, and water. The image pairs in this dataset are coarsely registered using a global calibration method; so, pixel-level registration could not be guaranteed. We cropped each of the nine types of scene images to 256 × 256 and did a mirror flip. Then, we selected two types of scene images, fields, and streets, to merge as the training set and test set of the experiment, for a total of 5,616 RGB-NIR image pairs. Among them, 5,460 image pairs were used as the training set and 156 were used as the test set.

### 3.2. Experimental environment

The basic configuration of the operating environment of the edge device is the same. The system is Ubuntu 18.04 for ARM, the Jet Pack version is 4.5, the CUDA version is 10.2, the cuDNN version is 8.0.0, the OpenCV version is 4.1.1, and the TensorRT version is 7.1.3. The selected configuration is currently more stable because different system versions and dependent environments impact device performance.

### 3.3. Subjective assessment

**FIGURE 14:**
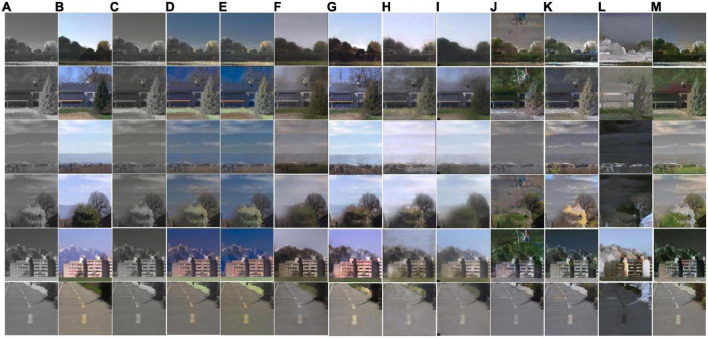
Subjective comparison of near-infrared (NIR) image colorization effects of different models on RTX3060. **(A)** Input; **(B)** label; **(C)** CICZ (Zhang et al., 2016); **(D)** ELGL (Iizuka et al., 2016); **(E)** ChromaGAN (Vitoria et al., 2020); **(F)** SCGAN (Zhao et al., 2020); **(G)** Pix2Pix (Isola et al., 2017); **(H)** MemoPainter (Yoo et al., 2019); **(I)** TIC-CGAN (Kuang et al., 2020); **(J)** CycleGAN (Zhu et al., 2017); **(K)** RecycleGAN (Bansal et al., 2018); **(L)** PearlGAN (Luo et al., 2022); **(M)** I2V-GAN (Li et al., 2021).

As shown in Figure 14, different methods perform quite differently on the RGB-NIR scene dataset used in this work. Pix2Pix has the best image effect, closest to the visible image, as shown in Figure 14G. TIC-CGAN’s performance is slightly blurrier than that of Pix2Pix. The image effect of the MemoPainter is different from the color of the visible image, and the image effect of SCGAN is dark. The subjective evaluation of the models based on Cycle-Consistent Adversarial Networks (CycleGAN, RecycleGAN, PearlGAN, I2V-GAN) is poor, especially in the images shown in Figure 14L. The reason is that the number of training sets is small, and the network cannot learn representative features. The CICZ does not learn helpful information on the datasets used in this work, resulting in subjective evaluation close to NIR images, as shown in Figure 14C. ELGL and ChromaGAN directly combine the L-channels of the NIR image during colorization to preserve details but with severe color deviations.

### 3.4. Objective assessment

#### 3.4.1. Image quality

We found that in the 11 models tested, the results of their image quality metrics on different edge devices were the same with only a few subtle differences; so, we only compared the test results on the RT3060 device. As shown in Table 1, from the image quality metrics, PSNR and SSIM, Pix2Pix, and TIC-CGAN have the best results, followed by MemoPainter. Part of the reason for the poor performance of CNN methods is that they combine L-channels, the brightness of NIR images when they finally generate the colorized images. This results in a significant difference between them and visible images.

**TABLE 1:** Evaluation of different image colorization models based on Peak Signal to Noise Ratio (PSNR) and Structural Similarity (SSIM) on Jetson AGX Xavier, Jetson Xavier NX, Jetson Nano, and RTX3060 devices.

Devices	RTX3060	AGX	NX	Nano
Method	PSNR/SSIM			
CICZ	14.249/0.565	14.265/0.565	14.265/0.565	14.265/0.565
ELGL	14.834/0.572	14.806/0.572	14.834/0.572	14.834/0.572
ChromaGAN	14.902/0.569	14.902/0.570	14.696/0.564	14.902/0.570
SCGAN	16.714/0.621	16.714/0.621	16.714/0.621	16.714/0.621
Pix2Pix	22.140/0.580	22.139/0.580	22.122/0.579	22.122/0.580
MemoPainter	18.645/0.535	18.645/0.535	18.645/0.535	–
TIC-CGAN	20.589/0.642	20.590/0.642	20.590/0.642	20.590/0.642
CycleGAN	14.139/0.535	14.139/0.535	14.139/0.535	14.139/0.535
RecycleGAN	14.083/0.474	14.087/0.472	14.098/0.472	14.087/0.471
PearlGAN	13.548/0.475	13.536/0.474	13.536/0.474	13.536/0.474
I2V-GAN	13.637/0.485	13.614/0.485	13.599/0.484	13.631/0.484

#### 3.4.2. Resource occupancy

As shown in Table 2, the latency and the inference speed of the 11 compared models vary significantly across different edge devices. We found that, as the performance of the devices decreases, the ratio of latency difference to each other also narrows. ELGL (46.8 FPS), Pix2Pix (45.0 FPS), CycleGAN (44.9 FPS), ChromaGAN (28.4 FPS), and TIC-CGAN (22.7 FPS) achieve real-time colorization on the Jetson AGX Xavier. Pix2Pix (27.4 FPS), CycleGAN (26.4 FPS), and ELGL (23.4 FPS) can achieve real-time colorization on the Jetson Xavier NX. The fastest on the Jetson Nano is Pix2Pix (6.8 FPS), followed by CycleGAN (6.3 FPS). We found that the fastest model to run on high-performance devices does not necessarily represent the fastest model to run on low-performance devices. Combined with the data in Figure 14, we believe that the running speed of a model with larger memory usage may be significantly affected when the memory resources are limited.

**TABLE 2:** Evaluation of different image colorization models based on latency and Frames Per Second (FPS) on Jetson AGX Xavier, Jetson Xavier NX, and Jetson Nano.

Devices	AGX	NX	Nano	AGX	NX	Nano
Method	Latency (s)			FPS		
CICZ	0.157	0.263	0.831	6.370	3.801	1.203
ELGL	0.021	0.043	0.191	46.823	23.397	5.244
ChromaGAN	0.035	0.072	0.270	28.387	13.805	3.703
SCGAN	0.159	0.303	1.221	6.305	3.301	0.819
Pix2Pix	0.022	0.036	0.146	44.986	27.426	6.863
MemoPainter	0.063	0.109	–	15.806	9.164	–
TIC-CGAN	0.044	0.081	0.412	22.731	12.288	2.427
CycleGAN	0.022	0.038	0.157	44.900	26.395	6.351
RecycleGAN	0.250	0.433	2.291	4.006	2.309	0.436
PearlGAN	0.147	0.252	1.058	6.799	3.968	0.945
I2V-GAN	0.226	0.388	2.051	4.433	2.574	0.488

**FIGURE 15:**
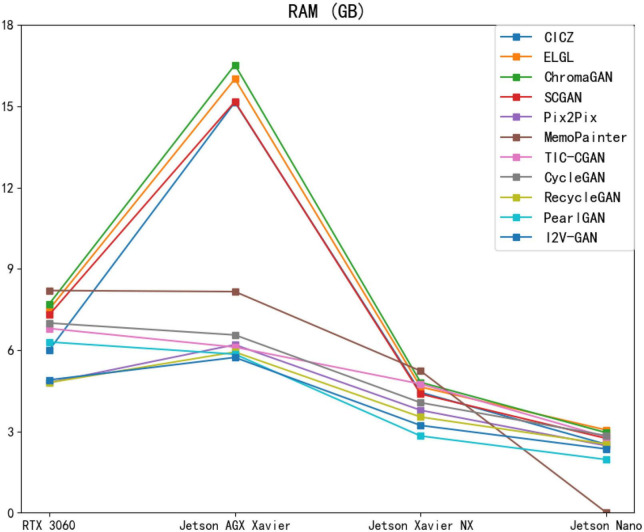
Performance of different image colorization models on Jetson AGX Xavier, Jetson Xavier NX, Jetson Nano, and RTX3060 devices for RAM metrics.

The initial memory usage of the server is 7.2 GB, the initial memory usage of Jetson AGX Xavier is 0.72 GB, the initial memory usage of Jetson Xavier NX is 0.56 GB, and the initial memory usage of Jetson Nano is 0.52 GB. The RAM values in Table 3 are the measured values minus the initial memory usage. As shown in Table 3, Figure 15, when the model is deployed on edge devices with sufficient running memory, it will occupy more than those with limited memory. This phenomenon may be related to the memory invocation principle of the PyTorch framework. I2V-GAN consumes the least memory on Jetson AGX Xavier. PearlGAN consumes the least memory on Jetson Xavier NX and Jetson Nano. MemoPainter cannot be run on Jetson Nano due to excessive memory usage.

**TABLE 3:** Evaluation of different image colorization models based on RAM and maximum current (Imax) on Jetson AGX Xavier, Jetson Xavier NX, and Jetson Nano.

Devices	AGX	NX	Nano	AGX	NX	Nano
Method	RAM (GB)			Imax (A)		
CICZ	15.150	4.440	2.520	1.370	0.650	1.460
ELGL	16.010	4.640	3.050	1.840	0.860	1.510
ChromaGAN	16.530	4.810	2.950	1.970	0.890	1.650
SCGAN	15.170	4.380	2.740	1.980	0.910	1.710
Pix2Pix	6.200	3.780	2.470	1.630	0.800	1.610
MemoPainter	8.160	5.230	–	1.680	0.770	–
TIC-CGAN	6.110	4.750	2.790	1.730	0.820	1.590
CycleGAN	6.560	4.060	2.840	1.650	0.810	1.600
RecycleGAN	5.930	3.530	2.530	1.770	0.840	1.610
PearlGAN	5.850	2.830	1.960	1.700	0.850	1.690
I2V-GAN	5.730	3.220	2.350	1.730	0.840	1.540

#### 3.4.3. Energy consumption

**FIGURE 16:**
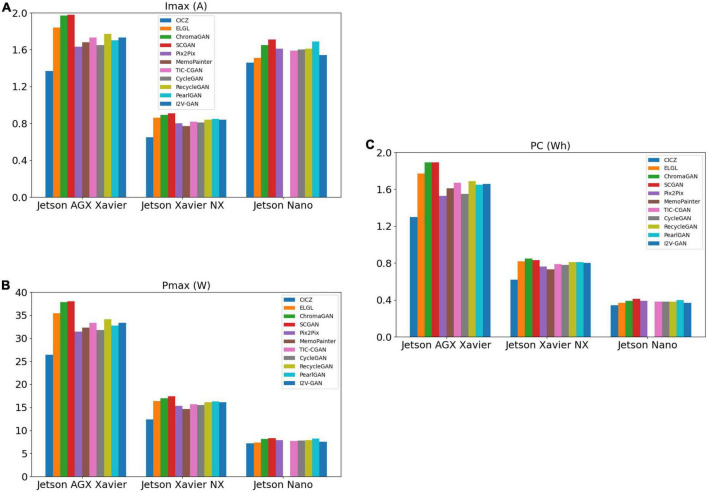
Performance of different image colorization models on Jetson AGX Xavier, Jetson Xavier NX, and Jetson Nano for maximum current (Imax) **(A)**, maximum power (Pmax) **(B)**, and power consumption (PC) **(C)**.

As shown in Figures 16B, C, the comparison model’s performance of the maximum power and total power consumption has the same trend. Since both Jetson AGX Xavier and Jetson Xavier NX are rated at 19 V and Jetson Nano is rated at 5 V, the maximum current of the model on Jetson Nano is higher than that on Jetson Xavier NX when the performance is limited, as shown in Figure 16A and Table 3. CICZ has the smallest energy consumption per unit time when it runs on the three edge devices, as shown in Table 4. The total power consumption is the power consumption in a certain period rather than the power consumption of each inference. Therefore, when selecting a model on an edge device with limited energy, we had to consider both the model’s latency (or FPS) and energy consumption metrics.

**TABLE 4:** Evaluation of different image colorization models based on maximum power (Pmax) and power consumption (PC) on Jetson AGX Xavier, Jetson Xavier NX, and Jetson Nano.

Devices	AGX	NX	Nano	AGX	NX	Nano
Method	Pmax (W)			PC (Wh)		
CICZ	26.400	12.400	7.200	1.300	0.620	0.340
ELGL	35.400	16.400	7.400	1.770	0.820	0.370
ChromaGAN	37.800	17.000	8.100	1.890	0.850	0.390
SCGAN	38.000	17.400	8.300	1.890	0.830	0.410
Pix2Pix	31.400	15.300	7.900	1.530	0.760	0.390
MemoPainter	32.300	14.600	–	1.610	0.730	–
TIC-CGAN	33.300	15.700	7.700	1.670	0.790	0.380
CycleGAN	31.800	15.500	7.800	1.550	0.780	0.380
RecycleGAN	34.100	16.100	7.900	1.690	0.810	0.380
PearlGAN	32.700	16.300	8.200	1.650	0.810	0.400
I2V-GAN	33.300	16.100	7.500	1.660	0.800	0.370

#### 3.4.4. Equilibrium assessment

**FIGURE 17:**
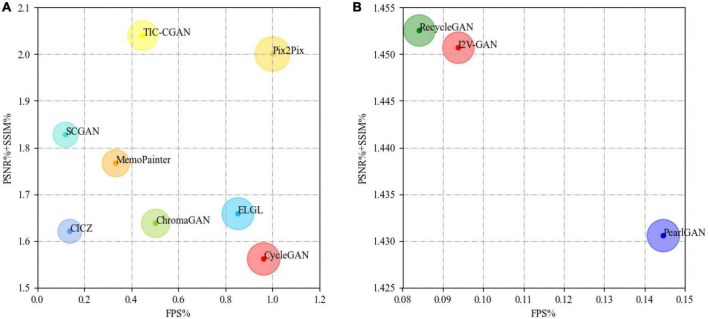
Comparison of different image colorization models [**(A)**: CICZ (Zhang et al., 2016), ELGL (Iizuka et al., 2016), ChromaGAN (Vitoria et al., 2020), SCGAN (Zhao et al., 2020), Pix2Pix (Isola et al., 2017), MemoPainter (Yoo et al., 2019), TIC-CGAN (Kuang et al., 2020), CycleGAN (Zhu et al., 2017); **(B)**: RecycleGAN (Bansal et al., 2018), PearlGAN (Luo et al., 2022), I2V-GAN (Li et al., 2021)]. The size of the circle represents the combined weight of the values on X-axis and Y-axis. The larger the circle, the better the performance.

From the results shown in Table 5, we believe that, if a model is suitable for running on edge devices, it requires a balance between the quality of colorized results and the inference speed. In general, on the edge device (Jetson Xavier NX), Pix2Pix can achieve real-time NIR image colorization requirements and has good image quality, as shown in Figure 17A. TIC-CGAN is slightly inferior in terms of latency. The performance differences between RecycleGAN, PearlGAN, and I2V-GAN are insignificant, as shown in Figure 17B.

**TABLE 5:** Evaluation of different image colorization models based on Peak Signal to Noise Ratio (PSNR), Structural Similarity (SSIM), and Frames Per Second (FPS) on Jetson Xavier NX.

Method	PSNR (%)	SSIM (%)	FPS (%)
CICZ	0.645	0.976	0.139
ELGL	0.671	0.988	0.853
ChromaGAN	0.664	0.974	0.503
SCGAN	0.756	1.072	0.120
Pix2Pix	1.000	1.000	1.000
MemoPainter	0.843	0.924	0.334
TIC-CGAN	0.931	1.109	0.448
CycleGAN	0.639	0.923	0.962
RecycleGAN	0.637	0.815	0.084
PearlGAN	0.612	0.819	0.145
I2V-GAN	0.615	0.836	0.094

This (%) represents the ratio of the model and Pix2Pix on the corresponding metric.

## 4. Conclusion

The Jetson series is a widely used embedded system. Limits on hardware resources and energy consumption restrict the deployment of current deep learning models on edge devices. In this study, an evaluation system was designed to test the performance of NIR image colorization methods on edge devices on the RGB-NIR scene dataset (Brown and Süsstrunk, 2011). From the experimental results, we summarize several conclusions for reference and provide suggestions for future work:

1. We found that the data were very close by comparing the results of the image quality metrics of the same model on the server and the edge devices. When considering image quality metrics of methods, researchers only needed to refer to the results on the server.
2. Among the 11 methods, the image quality metrics of Pix2Pix and TIC-CGAN were the best on the RGB-NIR scene dataset (Brown and Süsstrunk, 2011).
3. The latency of each model varied significantly across different edge devices. As device performance decreased, the proportion of the latency differences among the models also changed.
4. Of the 11 methods, ELGL had the smallest latency on Jetson AGX Xavier. On Jetson Xavier NX and Jetson Nano, Pix2Pix had the smallest latency.
5. When deployed on an edge device with enough running memory, the model will occupy more memory than the memory-limited device. The memory usage may be related to the memory allocation policy of the deep learning framework.
6. The RecycleGAN, PearlGAN, and I2V-GAN had smaller memory usage on edge devices than the others. Since we used only the generator to create colorized results for model testing, researchers who wish to optimize a model’s memory usage can refer to these models’ generator structures.
7. Of the 11 methods, CICZ had the smallest energy consumption per unit of time, while the maximum current and maximum power were the smallest. Meanwhile, the difference in energy consumption among other models was lower than the difference between CICZ and them. For optimizing energy consumption, researchers can refer to the structure of CICZ.
8. Combining the testing results of image quality and latency metrics, it can be concluded that Pix2Pix and TIC-CGAN could serve as a basis for further optimization of NIR image colorization on edge devices.

## Data availability statement

Publicly available datasets were analyzed in this study. This data can be found here: https://www.epfl.ch/labs/ivrl/research/downloads/rgb-nir-scene-dataset/.

## Author contributions

SS proposed the theory, conducted the experiment, and wrote the manuscript. XJ proposed the general idea of this theory. QJ and XJ supervised this work and revised the manuscript. WW, KL, and HC participated in the design and testing of the experimental process. PL, WZ, and SY discussed the theory. All authors contributed to the article and approved the submitted version.
